# Supplementary material for: Creatine Alleviates Doxorubicin-Induced Liver Damage by Inhibiting Liver Fibrosis, Inflammation, Oxidative Stress, and Cellular Senescence
Source: Nutrients. 2020 Dec 24;13(1):41. doi: 10.3390/nu13010041 (PMC7824063; doi:10.3390/nu13010041)
Supplement: Supplementary file 1 [file nutrients-13-00041-s001.pdf]

Supplementary Materials

Table S1. List of mRNA primers used in qRT-PCR

Target Gene	Type	Forward Sequence	Reverse Sequence
FN-1	mRNA	GGGAGAAGTTTGTGCATGGT	GTTGTGCCTGGGTAGGTCTG
ACTB	mRNA	GGTGGCACCACCATGTACC	GCAATGATCTTGATCTTCATGG
IL-1β1	mRNA	GCACAGTTCCCCAACTGGTA	TGTCCCGACCATTGCTGTTT
NF-κB	mRNA	AACGTGAACTCGGCAACTCT	AGCCTGGCTGTGCTAGTTTT
MCP	mRNA	CAGGTCTCTGTCACGCTTCT	GTAGTTCTCCAGCCGACTCA
NRF-2	mRNA	GCCAAGCAGGAGGAGTTCTT	GGGCAGTCGTATTTGACCCA

Table S2. Serum chemistry analysis.

Parameters	CTRL/SAL	2%Cr/SAL	4%/2%Cr/SAL	CTRL/DOX	2%Cr/DOX	4%/2%Cr/DOX
Liver Weight, g	11.6 ± 0.18 (n=6)	12.32 ±0.14 (n=6)	10.83±0.18 (n=6)	10.43±0 .13 (n = 7)	8.29 ±0.28 (n = 7)	9.498 ± 0.12 (n=5)
Body Weight, g	333.5 ±5.47 (n=6)	344.83 ± 6.68 (n=6)	352.83±3.40 (n=6)	305.14± 3.77 (n = 7)	296.57 ± 0.72 (n=6)	284.67 ± 2.63 (n=6)
ALT (IU/L)	421.75 ± 85.30 (n=4)	356.75 ± 48.1 (n=4)	182 ± 26.48 (n=4)	308.75± 43.57 (n=4)	319.5 ± 68.98 (n=4)	214.25 ±36.69 (n=4)
AST (IU/L)	1421.75 ± 265.11 (n=4)	1409 ± 207.12 (n=4)	587.5± 90.4 (n=4)	1790.75± 265.71 (n=4)	1663±- 178.99 (n=4)	807.25± 137.54 (n=4)
ALP (IU/L)	156.75 ± 5.33 (n=4)	196.75 ±8.4 (n=4)	192.5 ± 3.49 (n=4)	98.5 ±12.59 (n=4)	104 ± 14.83 (n=4)	74.25 ± 7.43 (n=4)
T-Bilirubin (mg/dL)	0.1 ± 0 (n=4)	0.075 ± 0.011 (n=4)	0.1 ± 0.011 (n=4)	0.1± 0.018 (n=4)	0.125 ± 0.027 (n=4)	0.1 ±0.02 (n=4)
Lipemia (mg/dl)	9.75 ±.45 (n=4)	12.75 ± .84 (n=4)	6.33 ± .51 (n=4)	17 ±1.57 (n=4)	12.25 ±.99 (n=4)	9.67 ± 1.12 (n=4)
